# 4-Methyl-2-oxo-2*H*-chromen-7-yl 4-meth­oxy­benzene­sulfonate

**DOI:** 10.1107/S1600536811049476

**Published:** 2011-11-30

**Authors:** Suman Sinha, Hasnah Osman, Habibah A. Wahab, Madhukar Hemamalini, Hoong-Kun Fun

**Affiliations:** a School of Pharmaceutical Sciences, Universiti Sains Malaysia, 11800 USM, Penang, Malaysia; b School of Chemical Sciences, Universiti Sains Malaysia, 11800 USM, Penang, Malaysia; c X-ray Crystallography Unit, School of Physics, Universiti Sains Malaysia, 11800 USM, Penang, Malaysia

## Abstract

In the title compound, C_17_H_14_O_6_S, the 2*H*-chromene ring is essentially planar, with a maximum deviation of 0.016 (1) Å. The dihedral angle between the 2*H*-chromene and the benzene rings is 54.61 (5)°. The C atom of the meth­oxy group is close to coplanar with its attached ring [deviation = 0.082 (2) Å]. In the crystal, mol­ecules are connected *via* C—H⋯O hydrogen bonds, forming sheets lying parallel to the *bc* plane. Weak C—H⋯π inter­actions are also observed.

## Related literature

For applications and properties of coumarin derivatives, see: Sinha *et al.* (2011 ▶); Valente *et al.* (2010) ▶; Radanyi *et al.* (2008 ▶); Han *et al.* (2005 ▶); Cheng *et al.* (2004 ▶). For further synthetic details, see: Fusegi *et al.* (2009 ▶).
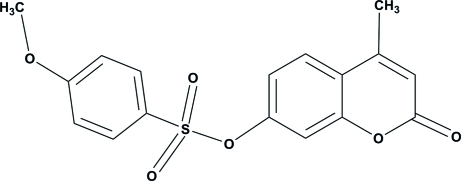


## Experimental

### 

#### Crystal data

C_17_H_14_O_6_S
*M* *_r_* = 346.34
Triclinic, 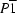

*a* = 7.9801 (3) Å
*b* = 9.2234 (4) Å
*c* = 10.9682 (5) Å
α = 99.049 (1)°
β = 90.288 (1)°
γ = 93.945 (1)°
*V* = 795.26 (6) Å^3^
*Z* = 2
Mo *K*α radiation
μ = 0.23 mm^−1^
*T* = 296 K
0.39 × 0.34 × 0.17 mm

#### Data collection

Bruker APEXII DUO CCD diffractometer
Absorption correction: multi-scan (*SADABS*; Bruker, 2009 ▶) *T*_min_ = 0.913, *T*_max_ = 0.962
21468 measured reflections
5907 independent reflections
4468 reflections with *I* > 2σ(*I*)
*R*_int_ = 0.022

#### Refinement

*R*[*F* ^2^ > 2σ(*F* ^2^)] = 0.041
*wR*(*F* ^2^) = 0.131
*S* = 1.04
5907 reflections
219 parameters
H-atom parameters constrained
Δρ_max_ = 0.34 e Å^−3^
Δρ_min_ = −0.36 e Å^−3^

### 

Data collection: *APEX2* (Bruker, 2009 ▶); cell refinement: *SAINT* (Bruker, 2009 ▶); data reduction: *SAINT*; program(s) used to solve structure: *SHELXTL* (Sheldrick, 2008 ▶); program(s) used to refine structure: *SHELXTL*; molecular graphics: *SHELXTL*; software used to prepare material for publication: *SHELXTL* and *PLATON* (Spek, 2009 ▶).

## Supplementary Material

Crystal structure: contains datablock(s) global, I. DOI: 10.1107/S1600536811049476/hb6524sup1.cif

Structure factors: contains datablock(s) I. DOI: 10.1107/S1600536811049476/hb6524Isup2.hkl

Supplementary material file. DOI: 10.1107/S1600536811049476/hb6524Isup3.cml

Additional supplementary materials:  crystallographic information; 3D view; checkCIF report

## Figures and Tables

**Table 1:** Hydrogen-bond geometry (Å, °) *Cg*1 is the centroid of the O2/C9–C13 ring.

*D*—H⋯*A*	*D*—H	H⋯*A*	*D*⋯*A*	*D*—H⋯*A*
C8—H8*A*⋯O3^i^	0.93	2.50	3.4156 (16)	169
C15—H15*A*⋯O5^ii^	0.93	2.44	3.2923 (17)	153
C16—H16*B*⋯*Cg*1^iii^	0.96	2.96	3.8423 (17)	154

Symmetry codes: (i) 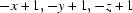; (ii) 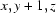; (iii) 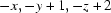.

**Fig. 1.**
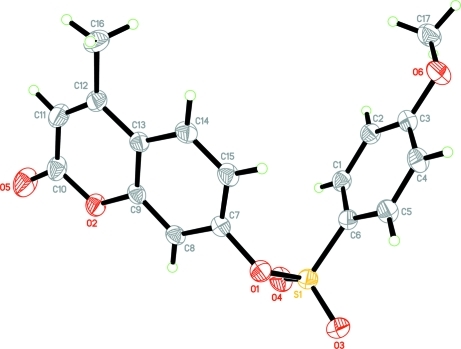
The asymmetric unit of the title compound, showing 30% probability displacement ellipsoids.

**Fig. 2.**
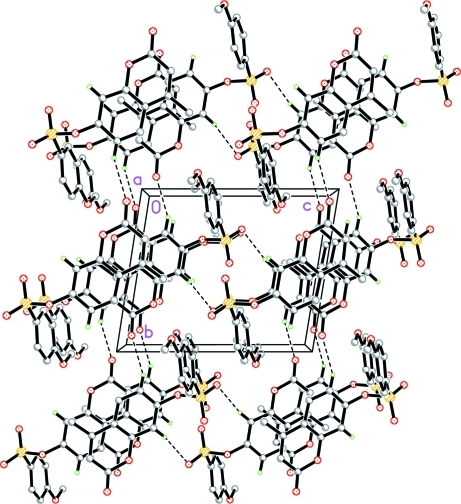
The crystal packing of the title compound (I). H atoms not involved in hydrogen bonding are omitted for clarity.

## supplementary crystallographic information

### Comment

This is the continuation of our work regarding synthesis of derivatives of sulphur-containing small molecules (Sinha *et al.*, 2011). Coumarin derivatives have been tested successfully against Cdc25 phosphatases (Valente *et al.*, 2010), HSP 90 (Radanyi *et al.*, 2008), MEK1 (Han *et al.*, 2005) as well as TNF-α (Cheng *et al.*, 2004). Apart from these biological activity, this class of molecules is also widely used as fluorescent labels for molecular studies of nucleic acids and proteins.

The asymmetric unit of the title compound is shown in Fig. 1. The 2*H*-chromene (O2/C7–C15) ring is essentially planar, with a maximum deviation of 0.016 (1) Å for atom O2. The dihedral angle between the 2*H*-chromene (O2/C7–C15) ring and benzene (C1–C6) ring is 54.61 (5)°.

In the crystal, (Fig. 2), the molecules are connected *via* weak intermolecular C—H···O hydrogen bonds (Table 1) to form two-dimensional networks parallel to the *bc*-plane.  Furthermore, the crystal structure is stabilized by weak C—H···π interactions involving the Cg1 (O2/C9–C13) ring.

### Experimental

The synthetic procedure followed is modified from the recent work by Fusegi *et al.* (2009). A mixture of 4-methylumbelliferone (0.176 g, 1.00 mmol), K_2_CO_3_ (0.345 g, 2.5 mmol), and 4 Methoxybenzene sulphonyl chloride (0.130 g, 1.1 mmol) in ethyl acetate (10 ml) was refluxed for 5 hrs. After cooling, the solvent was evaporated under reduced pressure. H_2_O (30 ml) was added to the residue and the contents was extracted with AcOEt (3 × 100 ml). The combined organic layer was washed with H_2_O (3 × 80 ml) and brine (1 × 100 ml) and was dried over MgSO_4_. The solvent was evaporated *in vacuo* and the residue was recrystallized from hexane - AcOEt (7: 1) to give the title compound as colourless blocks.

### Refinement

All hydrogen atoms were positioned geometrically [C–H = 0.93 or 0.96 Å] and were refined using a riding model, with *U*_iso_(H) = 1.2 or 1.5 *U*_eq_(C).  A rotating group model was applied to the methyl groups.

### Crystal data

**Table:**

C_17_H_14_O_6_S	*Z* = 2
*M**_r_* = 346.34	*F*(000) = 360
Triclinic, *P*1	*D*_x_ = 1.446 Mg m^−^^3^
Hall symbol: -P 1	Mo *K*α radiation, λ = 0.71073 Å
*a* = 7.9801 (3) Å	Cell parameters from 7512 reflections
*b* = 9.2234 (4) Å	θ = 2.6–32.8°
*c* = 10.9682 (5) Å	µ = 0.23 mm^−^^1^
α = 99.049 (1)°	*T* = 296 K
β = 90.288 (1)°	Block, colourless
γ = 93.945 (1)°	0.39 × 0.34 × 0.17 mm
*V* = 795.26 (6) Å^3^	

### Data collection

**Table:**

Bruker APEXII DUO CCD diffractometer	5907 independent reflections
Radiation source: fine-focus sealed tube	4468 reflections with *I* > 2σ(*I*)
graphite	*R*_int_ = 0.022
φ and ω scans	θ_max_ = 33.1°, θ_min_ = 2.2°
Absorption correction: multi-scan (*SADABS*; Bruker, 2009)	*h* = −11→11
*T*_min_ = 0.913, *T*_max_ = 0.962	*k* = −14→12
21468 measured reflections	*l* = −16→16

### Refinement

**Table:**

Refinement on *F*^2^	Primary atom site location: structure-invariant direct methods
Least-squares matrix: full	Secondary atom site location: difference Fourier map
*R*[*F*^2^ > 2σ(*F*^2^)] = 0.041	Hydrogen site location: inferred from neighbouring sites
*wR*(*F*^2^) = 0.131	H-atom parameters constrained
*S* = 1.04	*w* = 1/[σ^2^(*F*_o_^2^) + (0.0651*P*)^2^ + 0.114*P*] where *P* = (*F*_o_^2^ + 2*F*_c_^2^)/3
5907 reflections	(Δ/σ)_max_ < 0.001
219 parameters	Δρ_max_ = 0.34 e Å^−^^3^
0 restraints	Δρ_min_ = −0.36 e Å^−^^3^

### Special details

**Table:**

Geometry. All s.u.'s (except the s.u. in the dihedral angle between two l.s. planes) are estimated using the full covariance matrix. The cell s.u.'s are taken into account individually in the estimation of s.u.'s in distances, angles and torsion angles; correlations between s.u.'s in cell parameters are only used when they are defined by crystal symmetry. An approximate (isotropic) treatment of cell s.u.'s is used for estimating s.u.'s involving l.s. planes.
Refinement. Refinement of F^2^ against ALL reflections. The weighted R-factor wR and goodness of fit S are based on F^2^, conventional R-factors R are based on F, with F set to zero for negative F^2^. The threshold expression of F^2^ > 2σ(F^2^) is used only for calculating R-factors(gt) etc. and is not relevant to the choice of reflections for refinement. R-factors based on F^2^ are statistically about twice as large as those based on F, and R- factors based on ALL data will be even larger.

### Fractional atomic coordinates and isotropic or equivalent isotropic displacement parameters (Å^2^)

**Table:**

	*x*	*y*	*z*	*U*_iso_*/*U*_eq_	
S1	0.33642 (4)	0.69504 (3)	0.53507 (3)	0.04487 (10)	
O1	0.44708 (11)	0.69936 (10)	0.65864 (8)	0.04511 (19)	
O2	0.33520 (13)	0.28756 (9)	0.86131 (8)	0.0501 (2)	
O3	0.45083 (14)	0.76349 (12)	0.45924 (10)	0.0622 (3)	
O4	0.26867 (14)	0.54843 (11)	0.49601 (9)	0.0583 (3)	
O5	0.2954 (2)	0.08977 (12)	0.94880 (12)	0.0839 (4)	
O6	−0.18253 (12)	1.08511 (11)	0.72509 (10)	0.0568 (2)	
C1	0.01468 (16)	0.74728 (13)	0.59708 (11)	0.0438 (2)	
H1A	−0.0095	0.6464	0.5755	0.053*	
C2	−0.11060 (16)	0.83699 (14)	0.64295 (12)	0.0449 (2)	
H2A	−0.2193	0.7970	0.6513	0.054*	
C3	−0.07179 (15)	0.98751 (13)	0.67639 (11)	0.0419 (2)	
C4	0.08909 (16)	1.04825 (13)	0.65845 (13)	0.0469 (3)	
H4A	0.1127	1.1494	0.6781	0.056*	
C5	0.21342 (16)	0.96007 (14)	0.61205 (12)	0.0453 (3)	
H5A	0.3207	1.0008	0.5999	0.054*	
C6	0.17587 (15)	0.80817 (13)	0.58340 (10)	0.0405 (2)	
C7	0.37903 (14)	0.63892 (12)	0.75909 (10)	0.0386 (2)	
C8	0.38320 (15)	0.49001 (12)	0.75940 (10)	0.0396 (2)	
H8A	0.4218	0.4280	0.6918	0.048*	
C9	0.32790 (14)	0.43633 (12)	0.86381 (10)	0.0382 (2)	
C10	0.2809 (2)	0.21991 (15)	0.95863 (13)	0.0563 (3)	
C11	0.2140 (2)	0.31206 (16)	1.06288 (13)	0.0568 (3)	
H11A	0.1724	0.2679	1.1282	0.068*	
C12	0.20871 (17)	0.45853 (14)	1.07064 (11)	0.0458 (3)	
C13	0.26811 (14)	0.52666 (12)	0.96685 (10)	0.0383 (2)	
C14	0.26642 (17)	0.67727 (13)	0.96096 (11)	0.0452 (3)	
H14A	0.2273	0.7400	1.0279	0.054*	
C15	0.32143 (17)	0.73451 (13)	0.85815 (12)	0.0456 (3)	
H15A	0.3200	0.8346	0.8552	0.055*	
C16	0.1440 (2)	0.55079 (18)	1.18327 (12)	0.0630 (4)	
H16A	0.1057	0.4884	1.2412	0.095*	
H16B	0.0523	0.6035	1.1600	0.095*	
H16C	0.2324	0.6196	1.2206	0.095*	
C17	−0.34930 (19)	1.03035 (18)	0.74647 (17)	0.0627 (4)	
H17A	−0.4167	1.1113	0.7733	0.094*	
H17B	−0.3472	0.9685	0.8091	0.094*	
H17C	−0.3963	0.9743	0.6714	0.094*	

### Atomic displacement parameters (Å^2^)

**Table:**

	*U*^11^	*U*^22^	*U*^33^	*U*^12^	*U*^13^	*U*^23^
S1	0.05261 (18)	0.04271 (16)	0.04015 (15)	0.00954 (12)	0.00695 (11)	0.00638 (11)
O1	0.0427 (4)	0.0443 (4)	0.0496 (4)	0.0032 (3)	0.0038 (3)	0.0114 (3)
O2	0.0732 (6)	0.0319 (4)	0.0456 (4)	0.0135 (4)	0.0032 (4)	0.0030 (3)
O3	0.0705 (7)	0.0656 (6)	0.0567 (6)	0.0191 (5)	0.0255 (5)	0.0214 (5)
O4	0.0723 (7)	0.0449 (5)	0.0543 (5)	0.0094 (4)	−0.0045 (5)	−0.0048 (4)
O5	0.1473 (13)	0.0369 (5)	0.0711 (7)	0.0192 (6)	0.0102 (8)	0.0137 (5)
O6	0.0477 (5)	0.0450 (5)	0.0759 (7)	0.0090 (4)	0.0043 (5)	0.0016 (4)
C1	0.0511 (6)	0.0349 (5)	0.0454 (6)	−0.0002 (4)	0.0022 (5)	0.0075 (4)
C2	0.0427 (6)	0.0412 (6)	0.0511 (6)	−0.0009 (4)	0.0028 (5)	0.0102 (5)
C3	0.0430 (6)	0.0389 (5)	0.0441 (5)	0.0059 (4)	−0.0030 (4)	0.0061 (4)
C4	0.0463 (6)	0.0352 (5)	0.0579 (7)	0.0003 (4)	−0.0065 (5)	0.0040 (5)
C5	0.0402 (6)	0.0405 (6)	0.0553 (7)	0.0000 (4)	−0.0021 (5)	0.0089 (5)
C6	0.0437 (6)	0.0388 (5)	0.0397 (5)	0.0051 (4)	0.0012 (4)	0.0078 (4)
C7	0.0389 (5)	0.0359 (5)	0.0413 (5)	0.0057 (4)	−0.0001 (4)	0.0056 (4)
C8	0.0437 (6)	0.0355 (5)	0.0391 (5)	0.0105 (4)	0.0016 (4)	0.0007 (4)
C9	0.0439 (5)	0.0312 (4)	0.0389 (5)	0.0093 (4)	−0.0034 (4)	0.0006 (4)
C10	0.0830 (10)	0.0385 (6)	0.0486 (7)	0.0097 (6)	−0.0028 (6)	0.0090 (5)
C11	0.0819 (10)	0.0480 (7)	0.0427 (6)	0.0087 (6)	0.0026 (6)	0.0121 (5)
C12	0.0549 (7)	0.0465 (6)	0.0354 (5)	0.0081 (5)	−0.0028 (5)	0.0029 (4)
C13	0.0436 (5)	0.0361 (5)	0.0342 (5)	0.0079 (4)	−0.0033 (4)	0.0000 (4)
C14	0.0578 (7)	0.0358 (5)	0.0400 (5)	0.0114 (5)	0.0008 (5)	−0.0039 (4)
C15	0.0572 (7)	0.0309 (5)	0.0480 (6)	0.0087 (5)	0.0001 (5)	0.0011 (4)
C16	0.0873 (11)	0.0628 (9)	0.0383 (6)	0.0128 (8)	0.0089 (6)	0.0021 (6)
C17	0.0479 (7)	0.0606 (9)	0.0819 (10)	0.0127 (6)	0.0117 (7)	0.0141 (7)

### Geometric parameters (Å, °)

**Table:**

S1—O3	1.4203 (10)	C7—C8	1.3766 (15)
S1—O4	1.4210 (10)	C7—C15	1.3887 (16)
S1—O1	1.6076 (10)	C8—C9	1.3801 (16)
S1—C6	1.7401 (12)	C8—H8A	0.9300
O1—C7	1.4061 (14)	C9—C13	1.4011 (14)
O2—C9	1.3734 (13)	C10—C11	1.441 (2)
O2—C10	1.3760 (17)	C11—C12	1.3439 (19)
O5—C10	1.2018 (16)	C11—H11A	0.9300
O6—C3	1.3563 (15)	C12—C13	1.4509 (17)
O6—C17	1.4244 (18)	C12—C16	1.5011 (18)
C1—C6	1.3862 (17)	C13—C14	1.4017 (16)
C1—C2	1.3875 (18)	C14—C15	1.3796 (18)
C1—H1A	0.9300	C14—H14A	0.9300
C2—C3	1.3919 (17)	C15—H15A	0.9300
C2—H2A	0.9300	C16—H16A	0.9600
C3—C4	1.3924 (18)	C16—H16B	0.9600
C4—C5	1.3751 (18)	C16—H16C	0.9600
C4—H4A	0.9300	C17—H17A	0.9600
C5—C6	1.3979 (16)	C17—H17B	0.9600
C5—H5A	0.9300	C17—H17C	0.9600
			
O3—S1—O4	120.27 (7)	O2—C9—C8	115.88 (9)
O3—S1—O1	101.84 (6)	O2—C9—C13	121.43 (10)
O4—S1—O1	108.68 (6)	C8—C9—C13	122.69 (10)
O3—S1—C6	111.00 (6)	O5—C10—O2	116.70 (13)
O4—S1—C6	109.96 (6)	O5—C10—C11	126.35 (14)
O1—S1—C6	103.45 (5)	O2—C10—C11	116.95 (11)
C7—O1—S1	120.22 (7)	C12—C11—C10	123.40 (13)
C9—O2—C10	121.61 (10)	C12—C11—H11A	118.3
C3—O6—C17	118.18 (11)	C10—C11—H11A	118.3
C6—C1—C2	119.95 (11)	C11—C12—C13	118.25 (11)
C6—C1—H1A	120.0	C11—C12—C16	121.49 (12)
C2—C1—H1A	120.0	C13—C12—C16	120.26 (12)
C1—C2—C3	119.29 (11)	C9—C13—C14	117.23 (10)
C1—C2—H2A	120.4	C9—C13—C12	118.30 (10)
C3—C2—H2A	120.4	C14—C13—C12	124.45 (10)
O6—C3—C2	124.50 (11)	C15—C14—C13	121.51 (10)
O6—C3—C4	115.21 (11)	C15—C14—H14A	119.2
C2—C3—C4	120.28 (11)	C13—C14—H14A	119.2
C5—C4—C3	120.64 (11)	C14—C15—C7	118.37 (11)
C5—C4—H4A	119.7	C14—C15—H15A	120.8
C3—C4—H4A	119.7	C7—C15—H15A	120.8
C4—C5—C6	118.91 (11)	C12—C16—H16A	109.5
C4—C5—H5A	120.5	C12—C16—H16B	109.5
C6—C5—H5A	120.5	H16A—C16—H16B	109.5
C1—C6—C5	120.83 (11)	C12—C16—H16C	109.5
C1—C6—S1	120.13 (9)	H16A—C16—H16C	109.5
C5—C6—S1	119.00 (9)	H16B—C16—H16C	109.5
C8—C7—C15	122.74 (11)	O6—C17—H17A	109.5
C8—C7—O1	119.03 (10)	O6—C17—H17B	109.5
C15—C7—O1	118.06 (10)	H17A—C17—H17B	109.5
C7—C8—C9	117.46 (10)	O6—C17—H17C	109.5
C7—C8—H8A	121.3	H17A—C17—H17C	109.5
C9—C8—H8A	121.3	H17B—C17—H17C	109.5
			
O3—S1—O1—C7	−179.12 (8)	O1—C7—C8—C9	174.84 (10)
O4—S1—O1—C7	−51.20 (10)	C10—O2—C9—C8	−178.76 (12)
C6—S1—O1—C7	65.63 (9)	C10—O2—C9—C13	1.40 (19)
C6—C1—C2—C3	−0.89 (19)	C7—C8—C9—O2	−179.45 (10)
C17—O6—C3—C2	1.5 (2)	C7—C8—C9—C13	0.38 (18)
C17—O6—C3—C4	−179.69 (13)	C9—O2—C10—O5	−178.61 (15)
C1—C2—C3—O6	−178.23 (12)	C9—O2—C10—C11	0.7 (2)
C1—C2—C3—C4	3.04 (19)	O5—C10—C11—C12	176.67 (18)
O6—C3—C4—C5	178.64 (12)	O2—C10—C11—C12	−2.6 (2)
C2—C3—C4—C5	−2.5 (2)	C10—C11—C12—C13	2.2 (2)
C3—C4—C5—C6	−0.2 (2)	C10—C11—C12—C16	−177.60 (15)
C2—C1—C6—C5	−1.80 (19)	O2—C9—C13—C14	179.63 (11)
C2—C1—C6—S1	175.99 (9)	C8—C9—C13—C14	−0.20 (18)
C4—C5—C6—C1	2.33 (19)	O2—C9—C13—C12	−1.81 (17)
C4—C5—C6—S1	−175.49 (10)	C8—C9—C13—C12	178.37 (11)
O3—S1—C6—C1	145.04 (11)	C11—C12—C13—C9	0.03 (19)
O4—S1—C6—C1	9.50 (12)	C16—C12—C13—C9	179.81 (12)
O1—S1—C6—C1	−106.43 (10)	C11—C12—C13—C14	178.48 (13)
O3—S1—C6—C5	−37.13 (12)	C16—C12—C13—C14	−1.7 (2)
O4—S1—C6—C5	−172.67 (10)	C9—C13—C14—C15	−0.06 (18)
O1—S1—C6—C5	71.40 (10)	C12—C13—C14—C15	−178.53 (12)
S1—O1—C7—C8	83.63 (12)	C13—C14—C15—C7	0.1 (2)
S1—O1—C7—C15	−100.99 (12)	C8—C7—C15—C14	0.1 (2)
C15—C7—C8—C9	−0.32 (18)	O1—C7—C15—C14	−175.12 (11)

### Hydrogen-bond geometry (Å, °)

**Table:**

Cg1 is the centroid of the O2/C9–C13 ring.

**Table:**

*D*—H···*A*	*D*—H	H···*A*	*D*···*A*	*D*—H···*A*
C8—H8A···O3^i^	0.93	2.50	3.4156 (16)	169
C15—H15A···O5^ii^	0.93	2.44	3.2923 (17)	153
C16—H16B···Cg1^iii^	0.96	2.96	3.8423 (17)	154

Symmetry codes: (i) −*x*+1, −*y*+1, −*z*+1; (ii) *x*, *y*+1, *z*; (iii) −*x*, −*y*+1, −*z*+2.
